# Childhood health and the changing distribution of foreign aid: Evidence from Nigeria's transition to lower-middle-income status

**DOI:** 10.1371/journal.pone.0241866

**Published:** 2020-11-04

**Authors:** Carrie B. Dolan, McKinley Saunders, Ariel BenYishay

**Affiliations:** 1 Department of Health Sciences, William and Mary, Williamsburg, Virginia, United States of America; 2 Center for Health Analytics, Media, and Policy, RTI International, Raleigh, North Carolina, United States of America; 3 Department of Economics, William and Mary, Williamsburg, Virginia, United States of America; University of Glasgow, UNITED KINGDOM

## Abstract

With sustained economic growth in many parts of the developing world, an increasing number of countries are transitioning away from the most subsidized development finance as they exceed income and other qualification requirements. Cross-country evidence suggests that Development Assistance Committee (DAC) donors view the crossing over of the World Bank's International Development Association (IDA) eligibility threshold to signal that a country needs less aid, with subsequent reductions in both IDA and other donors' concessional funding. Within the health sector, it is particularly important to understand the implications of these status changes for children under five years of age since improving early childhood health is critical to fostering health and social and economic development. Therefore, we examine the implications of the IDA transition by measuring the extent t which World Bank commitments—including both IDA and IBRD—are directed to infant and child health needs in Nigeria. Ordinary Least Squares (OLS) models were used in a difference-in-differences (DID) strategy to compare World Bank IBRD/IDA lending before and after the crossover to regions with varying initial levels of under-five and infant need. We find that the infant need orientation of World Bank aid has increased post-crossover. Conversely, alignment of World Bank commitments to regional child needs appears to have diminished after Nigeria crosses the IDA threshold. However, these effects are statistically insignificant and therefore provides inconclusive evidence. This research addresses an important policy question because the transition away from concessional funding mechanisms will result in difficult tradeoffs in allocating limited health resources; without providing conclusive evidence that crossover results in changes in need-based allocation, it does offer an essential path for future research. These results are directly relevant to policy debates about what we know and do not know about aid in transition and health. This research's value is especially important in the Sustainable Development Goal (SDG) era in understanding how donor exits could derail progress in health improvement.

## Introduction

As the landscape of sustained economic growth continues to evolve in parts of the developing world, an increasing number of countries are transitioning away from the most subsidized development finance as they exceed income and other qualification requirements. Under current eligibility criteria, the World Bank's most concessional window, the International Development Association (IDA), will see significant transformations in its funding portfolio, which will shrink from 75 countries in 2015 to an estimated 25 countries in 2040 [1]. The World Bank shifts its support for such transitioning countries to its International Bank for Reconstruction and Development (IBRD), which provides financing at far less concessional terms. Thus, researchers and policymakers alike must devote additional attention to understanding the potentially difficult tradeoffs in allocating limited resources such a transition could entail.

Cross-country evidence suggests that Development Assistance Committee (DAC) donors view the World Bank's income categories as key markers of need (particularly in "low income" (LIC) and "lower middle income" (LMIC) categories). DAC donors also appear to view a country's crossing over the World Bank's International Development Association (IDA) eligibility threshold, related to a country's reclassification to LMIC status, to signal that a country needs less aid. This signal results in subsequent reductions in both IDA and other donors' concessional funding. Galiani et al. found an estimated 20% decline in official development assistance (ODA) after crossing the IDA income threshold. ODA is defined by the Organisation for Economic Co-operation and Development (OECD) as "government aid that promotes and specifically targets the economic development and welfare of developing countries" [2]. This decline may incentivize recipient countries to report data that underestimates per capita income for fear of losing concessional finance and aid allocation from other essential donors [3, 4]. Despite the anticipated adverse effects of decreases in concessional aid, there is very little quantitative evidence in the literature on this transition's effects. Of particular importance is understanding the significance of this status change within the health sector, where access gaps could have significant implications for key and vulnerable groups [5]. This sector is especially susceptible to eligibility changes since many large donors define eligibility based on income thresholds (c.f. Gavi, the Vaccine Alliance).

Though the Sustainable Development Goals (SDGs) are universal in scope to "leave no one behind" in their blueprint for a more sustainable future, there are 44 child-level indicators integrated across the 17 SDGs [6]. Progress towards these goals will depend on how global health actors navigate "diseases, demography, development assistance for health, and domestic health financing" [7]. Withdrawal of aid due to income status and graduation from concessional finance could substantially affect some countries' ability to progress towards these child-level goals. Moreover, geographic clustering of needs within countries may prove particularly challenging to address as concessional financing for health investments becomes scarcer. The academic and policy literature provides little systematic evidence on the implications of transitioning from LIC to LMIC at the sector level. The quantitative literature shows that as countries graduate, they gradually become more reliant on loans with more stringent terms and official finance conditions [8]. Since governments are less likely to borrow for social service projects with limited financial returns such as health or education, this funding is more likely to be channeled into economic infrastructure [2, 5]. Engen and Prizzon provide the first attempt to systematically examine the impact on development finance during donor transition. Through a case study approach, they examined eight countries that graduated from IDA between 1995 and 2012, finding that LMICs relied more on loans than grants. The sectoral allocation of resources shifted towards infrastructure development, such as transport and energy [2]. In Pakistan, which crossed the LIC to the LMIC threshold in 2009, the health sector accounted for 6% of aid in 2000 and only 1% in 2015 [2]. The government of Pakistan's domestic budget did not compensate for this reduction, with average domestic government health expenditure declining from 3.5% in the three years before the crossover to 3% in the most recent three years. These findings corroborate a concern that the health sector is vulnerable if resources are not directed to closing this gap [5]. This finding is especially concerning since the next cohort of graduates has more limited and less efficient health systems [7].

In sum, the empirical evidence is limited. Still, it suggests that a reduction in concessional finance likely results in changes in external and domestic funding sources' sectoral composition. Furthermore, recipient countries rarely have strategies to plan and coordinate the transition to mitigate the effects [2, 5, 8]. Notwithstanding this debate, there is no empirical literature on the impact of this sectoral shift on health outcomes, specifically in children under five.

It is particularly important to understand the implications of these status changes for children under five because gaps in assistance could have significant adverse health implications [4]. Although the literature has found improving early childhood health is critical to fostering health and social and economic development, [9] there is limited quantitative evidence in studying the implications of status changes for health aid and the resulting impact on young children's health. Several studies have investigated the effects of aid on public health expenditure, child vaccination, and child mortality, with results suggesting gains from aid investments and adverse effects from public sector reforms induced by IMF conditionality [10–12]. Kotsadam et al. (2018) studied how ODA affects aid effectiveness—as measured by infant mortality—at the subnational level in Nigeria with mother fixed-effects [10]. Using the Demographic and Health Surveys (DHS) data, Kotsadam et al. (2018) found that geographical proximity to active aid projects reduces infant mortality through a quasi-experimental approach. The researchers also found that aid contributes to a reduction in inter-group inequalities. However, the study also found that aid may cause adverse health outcomes in developing countries by failing to target the most at-risk populations.

In a series of papers on IMF conditionalities, Daoud and coauthors identified negative impacts on child health arising after the conditional reform programs are introduced. Daoud et al. (2017) found that IMF programs cause adverse effects in the rates of malnourishment, sanitation, shelter, and health care access, including immunizations in children. The study employed DHS data and was robust in variation and sample size [11]. In a time-series cross-sectional analysis of 128 developing countries from 1980–2014, Daoud and Reinsberg (2019) found that IMF policies, particularly those who require reforms of the public sector, undermine health in developing countries [12].

We examine the implications of the IDA transition by measuring the extent to which World Bank commitments—including both IDA and IBRD—are directed to infant and child health needs. We explore this directly using georeferenced data on the World Bank's activities and measures of underlying need at Nigeria's subnational region level. We focus on Nigeria for three reasons. First, losing access to concessional finance such as IDA could make it impossible to reach SDG-defined targets in newly vulnerable countries, such as Nigeria, Africa's most populous country. The World Bank classifies Nigeria as a blend country, a transitional designation meaning it maintains access to IDA funds but at a reduced scale [13, 14]. Second, in Nigeria, not only has ODA as a share of GDP fallen over time, so have tax revenues [2]. This challenge makes it difficult to generate meaningful population health gains as outlined in the SDGs [15]. Third, Nigeria is covered by several complementary data sources, including historical data from the World Bank, AidData, and the DHS.

This paper is the first to investigate World Bank crossover's role on a variety of infant and child health indicators of need at a subnational level. We specifically focus on the World Bank because research indicates that DAC donors view the operational cutoff for IDA eligibility to signal that a country is in less need of aid [2]. Research suggests that health programs tend to be supported by grants and concessional finance rather than non-concessional loans from donor governments [2]. Therefore, we expect that aid should decrease after crossing the income threshold regarding its child health focus. It is important to note that our contribution is not examining the effects this targeting has on infant and child health needs. Instead, we examine the extent to which allocation to infant and child health changes after the IDA transition. We investigate the question: Does the World Bank targeting of IDA and IBRD funding toward infant and child health needs a change after Nigeria's IDA transition?

### Conceptual framework

From our review of the literature, no commonly used framework explains donor motivation post-transition. However, from a broad perspective, research indicates that health programs tend to be supported by grants and concessional finance rather than non-concessional loans from donor governments [2]. In this research, we built on existing work by selecting need as an indicator to examine how IBRD/IDA assistance would react to the crossing of the income threshold in terms of a child's health focus. The need was selected because this indicator plays a significant role in shaping supply and demand for financing in financing for sustainable development markets [16, 17]. Need is often used to combine various metrics of interest to donors and determine whether or not a country can address critical issues if donor support were to change. There are a variety of definitions for the term need. We use the term to broadly categorize health outcomes and mortality burden based on the leading causes of death in Nigeria in 2003 [18]. The five health outcomes we selected serve as a proxy to identify "high risk" areas where aid may be targeted to help the most vulnerable populations. This "proxy" approach is employed throughout the literature and is similarly used in Kotsadam et al. (2018), where infant mortality is used to study aid effectiveness [10]. We hypothesize that an aggregate measure of these five health outcomes could be improved in either the short or intermediate run by targeted aid. The concept of need aligns with the newly developed OECD ABC methodology, which provides operational guidance for development partners to conduct transition finance analyses. This methodology focuses on key elements relevant to understanding transition finance through an assessment, benchmarking, and counseling approach. Our work aligns with the assessment phase of the methodology by examining the transition context and its implications [16].

## Materials and methods

**Fig 1:**
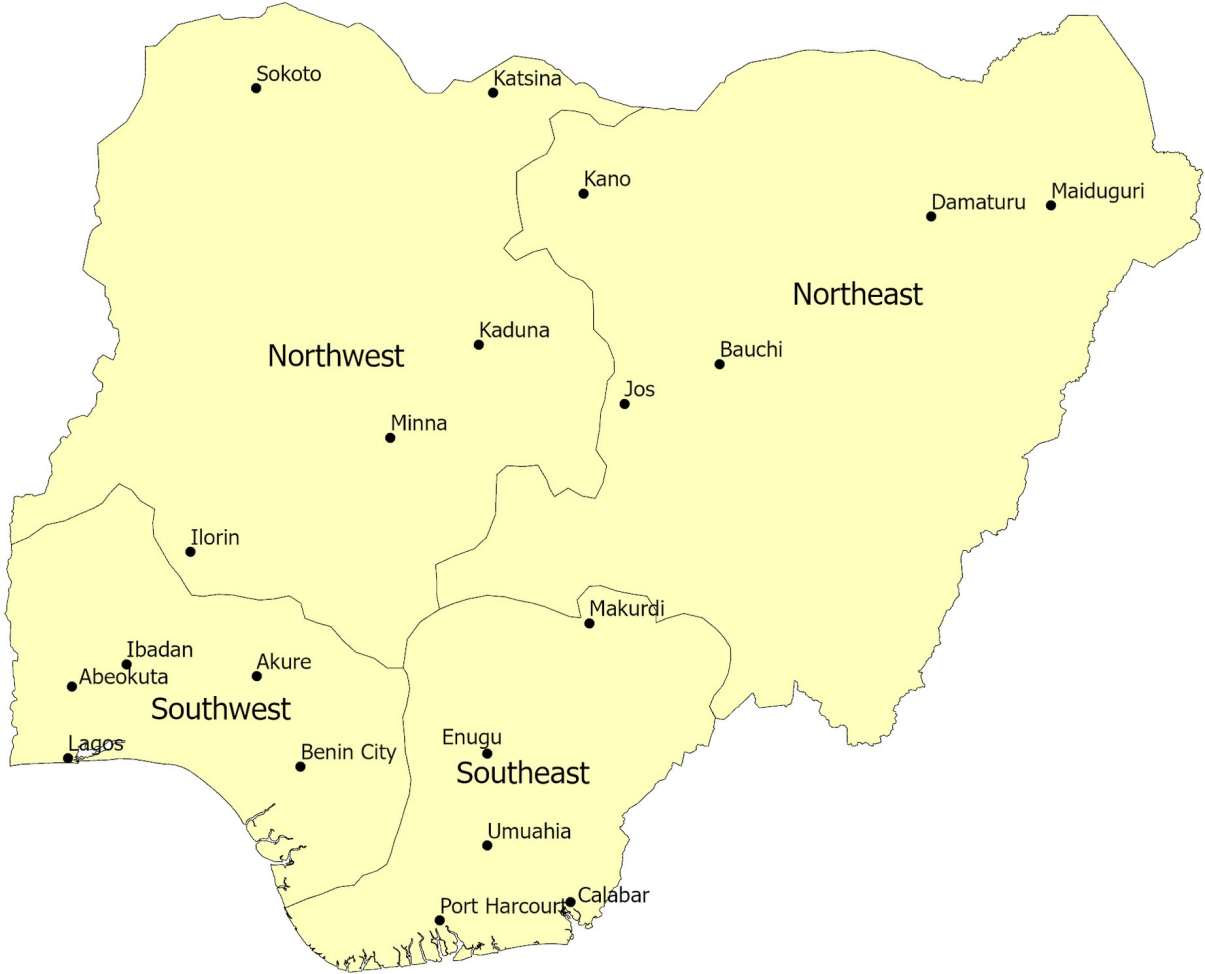
Nigeria subnational units defined by the Demographic Health Survey.

This section introduces the data and empirical strategy we use to test whether crossover matters for World Bank health aid allocation to infant and child health needs. Our sample covers four subnational units (Northeast, Southeast, Northwest, and Southwest) in Nigeria over the 1995–2014 time period (Fig 1). There are four regions over twenty years resulting in a region year panel (N = 80), which is the unit of observation for analysis. The DHS defined these broad geographic regions, which were selected because they provide consistent geographic boundaries over the study period. Careful consideration of the boundaries is vital to ensure the reliability of the results.

We aim to detect changes in the regional association of health aid and needs before and after Nigeria's crossing of the IDA income threshold. One potential challenge is that the regional measures of young children's health needs may endogenously respond to health aid changes, thereby confounding our estimates. For example, it is possible that, after the crossing, regions with worse initial health conditions saw increases in health aid, which subsequently improved health conditions in those regions, leaving the relationship between health aid and post-crossing health conditions similar to the pre-crossing relationship (despite greater aid being allocated to initially worse off regions). Therefore, we fix our health need measures based only on pre-crossing conditions, allowing us to observe any changes in aid distribution relative to these pre-crossing need measures. As described below, our difference-in-difference empirical strategy combines cross-sectional variation in pre-crossing needs and temporal variation across the pre- and post-crossing periods to generate.

To link time-varying aid data and fixed pre-crossing health need measures, we combined data from two sources: the 2003 Nigeria Demographic Health Survey [NDHS] and the 1995–2014 AidData WB IBRD-IDA, level 1, Version 1.4.2 dataset [AidData WB] [19, 20]. The 2003 NDHS serves as our primary source for five infant and child health indicators. Nigeria crossed the IDA income threshold in 2008, and the 2003 NDHS provides the most recent pre-crossing measure of conditions across Nigeria's regions.

From the 2003 NDHS, we utilized five indicators of infant and child health: neonatal mortality, under-five mortality and prevalence of diarrhea, acute respiratory infection, and/or fever in children under five. These health indicators consistently contribute to the leading causes of morbidity among children under five in Nigeria during the study period [18]. Also, we include overall measures of mortality burden. We rely on a respondent's complete childbirth and death history extracted from a nationally representative sample of women. These birth histories are often used to measure the mortality of children younger than the age of five in developing counties and look at child health indicators such as immunization coverage and recent occurrences of diarrhea, fever, and cough for young children. We then utilized the georeferenced data available from the 2003 NDHS by merging the birth recode files with the GPS data file at the child level to calculate the mortality measures, and by merging the children’s recode file to calculate the prevalence of ARI, diarrhea and fever, we could assign the interview site's specific latitude/longitude to one of four constant subnational units in Nigeria over 1995–2014. Finally, we calculated these measures regionally following the DHS Guide to Statistics (Table 1) [21].

**Table 1:** Regional measures of infant and child health determinants.

Health Indicator	Calculation	Data Source
Under-five child mortality	The direct estimation of under-five mortality rates is based on a synthetic cohort life table for successive five-year periods before the survey. Through this life table, we measured the cumulative probability of dying at the beginning of year five.	2003 NDHS; Birth recode file
Neonatal mortality	The percentage of children born in the five years preceding the survey who died within the first 30 days of birth, divided by the total number of live children born in the past five years.	2003 NDHS; Birth recode file
Prevalence of diarrhea in children under five	The percentage of children under five who had diarrhea in the two weeks preceding the survey across the sample, divided by all live births in the five years preceding the survey.	2003 NDHS; Children's recode file
Acute respiratory infection in children under five	The percentage of live children under five with symptoms of ARI—having a cough and short, rapid breathing—in the two weeks preceding the survey. We then divided this ARI calculation by all live births in the five years preceding the survey.	2003 NDHS; Children's recode file
Fever in children under five	The prevalence of fever is estimated by asking mothers whether their children under five had a fever in the two weeks preceding the survey. These results are divided by the total number of live, under-five children they report.	2003 NDHS; Children's recode file

We use the AidData World Bank IBRD-IDA, level 1, Version 1.4.2 dataset to construct our outcome measures, including all World Bank projects approved in 1995–2014 in the IBRD and IDA lending lines. The initial global data set consists of 5,881 projects and 61,243 project locations, comprising total commitments of US$645 billion and total disbursements of US$394 billion, which was subset to include health-specific projects totaling US$105.3 million (Table 2). AidData's dataset uses a coding system comparable to the OECD Creditor Reporting System, which assigns a standardized code for each project that reflects the sector targeted by the project. There are two health aid sectors in the subnational dataset: CRS Code 120 and 121 [22, 23]. This data includes both concessional (IDA) and non-concessional (IBRD) flows. The data set consists of a geocoding tag that provides the latitude and longitude of health-specific projects. These point-based locations were then assigned to the defined DHS subnational units of Northeast, Southeast, Northwest, and Southwest, resulting in a DHS region level total of aid commitments.

**Table 2:** Summary statistics in $US, World Bank Health aid, DHS region, pre-and post-crossover.

	Northeast	Northwest	Southeast	Southwest
**1995–2007**				
Mean	1,009,336	740,835	487,529	360,401
SD	1,269,647	975,104	615,530	437,366
Min	0	0	0	0
Max	3,522,013	3,050,478	1,931,298	1,333,109
Total	13,121,372	9,630,862	6,337,885	4,685,213
**2008–2014**				
Mean	4,358,992	2,218,785	1,781,075	1,853,717
SD	5,125,076	2,522,816	2,228,877	2,306,459
Min	0	0	0	0
Max	12,500,000	6,612,651	5,935,714	6,216,351
Total	30,530,706	15,531,494	12,467,525	12,976,020

### Indices of need

After regional level statistics were calculated, we applied principal components analysis (PCA) to develop relative indices of infant and under-five need based on five health indicators: neonatal mortality, under-five mortality, and percent of diarrhea, acute respiratory infection, and fever in children under five. Principal components analysis has been used extensively with population and health survey data to construct socioeconomic status measures [24]. It is a multivariate statistical technique for reducing a larger number of variables into a substantially smaller set of uncorrelated variables without losing information [25]. The use of variables measured on different scales can result in other variances. To minimize these differences, the PCA was performed using standardized variables. With PCA, factors are generated that describe the commonality of a set of related indicators. Factors were retained if the associated eigenvalue was greater than one. Rotation was used to produce orthogonal factors that are not correlated and facilitate the components' interpretation. The first component explained 69.0% of the total variability. It gave the greatest weight to the prevalence of diarrhea in children under five (.99), the prevalence of fever in children under five (.97), and under-five mortality rates (.95). For further analysis, we considered an additional principal component. The second principal component accounted for 22.5% of the variation in the original data. It showed that the weights were concentrated on neonatal mortality (.96) and prevalence of ARI symptoms in children under five (.43). Following principal components analysis, indices of need were predicted based on the scores from factors 1 and 2, defined as under five and infant needs. They were included as continuous independent variables in subsequent regression models.

### Crossover

Eligibility for IDA support depends on the country's relative poverty, defined as national GNI per capita below an established threshold and updated annually ($1,165 in the fiscal year 2018) [26]. Nigeria crossed over this income threshold in 2008 [27]. As noted above, it was then reclassified as a blend country, making it eligible for IDA programs but also creditworthy for some IBRD borrowing [28]. In practice, funding from IDA decreased after 2008, with IBRD funding partially (but not entirely) offsetting this decline. In this paper, *crossover* refers to Nigeria's exceeding the LMIC threshold and falling-out of IDA's full concessional window in 2008.

### Additional controls

We include region-level fixed effects that control unobserved time-invariant region characteristics associated with need and funding. Finally, we add year fixed effects to control for different funding trends across regions before crossover.

### Study design

We utilized a difference-in-differences approach to compare pre- and post-crossover variation in WB commitments across varying initial needs regions. We combined cross-sectional variation in pre-crossing need measures with temporal variation across the threshold crossing for identification in this approach. That is, our difference-in-differences approach compared regions' health aid based on their needs both before and after the crossover. One interpretation of such a design in program evaluation terminology is that lower-needs regions effectively serve as a comparison group for higher-needs regions, with baseline differences across these groups measured before the threshold crossing and post-treatment differences measured after the crossing. We include year fixed effects, which flexibly de-trend the funding data at the country level to prevent spurious correlation with the timing of the income threshold crossover and region fixed effects, absorbing time-invariant omitted variables that could be correlated with funding levels and initial conditions. This approach allows us to examine whether the relationship between aid and child health needs changes during such a transition. In doing so, we reflect only need conditions measured from the initial round of the DHS, as subsequent round measurements could endogenously reflect the effects of World Bank funding, thereby biasing our estimates of the relationship between conditions and aid funding.

### Empirical specification

Ordinary Least Squares (OLS) models were used in a difference-in-differences (DID) strategy to compare World Bank IBRD/IDA lending before and after the crossover to regions with varying initial levels of under-five and infant need. Our baseline regression model, estimated by OLS over the regions from 1995 through 2014, can be defined as follows:

$$WorldBan{k\_Commit}_{{}_{rt}}=\beta_{1}Crossover{}_{t}+\beta_{2}Under5Need{}_{r}+\beta_{3}InfantNeed{}_{r}+\beta_{4}(Crossover{}_{t}*Under5Need{}_{r})+\beta_{5}(Crossover{}_{t}*InfantNeed{}_{r})+\Theta_{t}+\Delta_{r}+\epsilon_{rt}$$

*WorldBank_Commit_rt_* is a continuous variable denoting World Bank health aid's total volume, including concessional (IDA) and non-concessional (IBRD) flows of region *r* in year *t* using a log scale. *Crossover_r_* is a binary variable equal to 1 in the year when the crossover occurs (2008) and subsequent years later. *β*_2_*Under*5*Need_r_* and *β*_3_*InfantNeed_r_* are continuous under-five and infant need measures, respectively, created from the PCA. The parameters of interest, *β*_4_, and *β*_5_ capture the differential relationship between need and World Bank commitments after the crossover. Θ_*t*_ is a year fixed effect to flexibly absorb national trends in aid and need that might confound our estimate. Δ_*r*_ is a region fixed effect that absorbs time-invariant characteristics of regions that might be correlated with World Bank commitments and need. The error term is clustered at the region-year level. All analysis was conducted using Stata, version 15.

## Results

### Descriptive statistics

Table 2 provides summary statistics of World Bank health aid per year at the DHS region level. Pre-crossover, the average region receives approximately US$649,526 in World Bank health aid compared to approximately US$2.5 million in the post-crossover period. At the region level, the average amount of aid was highest in the pre-crossover period in the Northeast (US$1m) region, followed by the Northwest (US$740,835), Southeast (US$487,529), and Southwest (US$360,401). In the post-crossover period, the average regional amounts of aid remained highest in the Northeast (US$4.3million) and Northwest (US$2.2m) but was slightly higher in the Southwest (US$1.9m) than the Southeast (US$1.8m). After regional level statistics were calculated (Table 3), we applied principal components analysis (PCA) to develop relative indices of infant and under-five need based on five health indicators: neonatal mortality, under-five mortality, and percent of diarrhea, acute respiratory infection, and fever in children under five.

**Table 3:** Descriptive statistics, DHS region, 2003.

	Northeast	Northwest	Southeast	Southwest
Under 5 Mortality Rate	23.58%	20.02%	17.29%	12.45%
Neonatal Mortality	3.54%	3.11%	3.75%	3.46%
Percent of Children Under 5 with Acute Respiratory Infection Symptoms	12.97%	8.36%	8.51%	9.66%
Percent of Children Under 5 with Diarrhea	29.09%	16.89%	10.16%	6.68%
Percent of Children Under 5 with a Fever	38.17%	30.51%	26.42%	19.19%

### Correlation of need, World Bank commitments, and crossover

The results of the DID analysis are shown in Table 4. We start by presenting our results to analyze aid allocation with year fixed effects to flexibly absorb national trends in aid and need that may confound our estimate (Columns 1–3). Subsequently, we also include a region fixed effect that absorbs time-invariant characteristics of regions that might be correlated with aid allocation and need (Columns 4–6). The maximum number of observations is 80 from 4 subnational regions over 20 years. However, not all of these observations contribute to the identification because 20 of these region years have no data for our dependent variable. The decrease in observations is not the result of the data generation process. Instead, it is a function that in the underlying dataset, some region-years get health aid, and others do not. Therefore, the coefficients are identified based on the variation from the remaining 60 observations. This specification finds that World Bank allocation was generally correlated with child needs across the period (Columns 1 and 4), and was less closely correlated with need after crossover; the latter effect is sizable but not statistically distinguishable from zero. Turning to infant need (Columns 2 and 5), we find that World Bank allocation was positively correlated with infant need once region fixed effects are accounted for (Column 5). This finding diminished post-crossover, but this effect was not statistically significant. The primary coefficients of interest were the interactions between crossover and child need as well as crossover and infant need. These results indicate that there was no significant correlations post-crossover when considering both child and infant needs (Columns 3 and 6).

**Table 4:** Crossover effects on World Bank health commitments.

	(1)	(2)	(3)	(4)	(5)	(6)
VARIABLES	OLS	OLS	OLS	OLS	OLS	OLS
Child Need	0.456***		0.455**	0.375**		0.518***
	(0.164)		(0.165)	(0.141)		(0.178)
Interaction: Crossover and Child Need	-0.203		-0.194	-0.181		-0.172
	(0.228)		(0.216)	(0.198)		(0.187)
Infant Need		-0.072	-0.067		2.164***	-1.119*
		(0.080)	(0.068)		(0.743)	(0.581)
Interaction: Crossover and Infant Need		0.205	0.214		0.220	0.213
		(0.172)	(0.160)		(0.146)	(0.146)
Constant	7.762***	8.041***	7.811***	8.081***	7.309***	8.540***
	(1.835)	(2.204)	(1.850)	(1.702)	(1.761)	(1.734)
Year FE	Y	Y	Y	Y	Y	Y
Region FE				Y	Y	Y
Observations	60	60	60	60	60	60
R-squared	0.945	0.926	0.947	0.952	0.952	0.953

The dependent variable is a continuous measure of World Bank IDA/IBRD health commitments on a log scale.

Robust standard errors in parentheses, clustered at the region-year level.

*** p<0.01

** p<0.05

* p<0.1.

### Sensitivity analysis

As a sensitivity analysis, we employed wild bootstrap to test the standard errors' robustness to adjust for the small number of region clusters (using cgmwildboot [29]). The adjustment did not alter the statistical significance of any of the results. Some readers might be rightfully concerned that the limited sample size threatens the reliability of our results. Therefore, we test whether post-crossover health aid flows were targeted to overall population measures of neonatal, infant, and child mortality at the ADM1 (state) level. To test this relationship, we leveraged the AidData GeoQuery tool (http://geo.aiddata.org/query) to extract an ADM1 year panel of "Health" and "Health, general" aid data from the World Bank Donor System, geocoded and published by AidData [30]. This data was merged with the newly released data set from the Institute for Health Metrics and Evaluation (IHME) of low and middle-income country neonatal, infant, and under-5 mortality geospatial estimates 2000–2017 to construct an ADM1 year panel of WB health aid and population levels (the year 2000, counts) measures of neonatal, infant, and child health (n = 555) [31]. We subsequently repeated the empirical approach of OLS models used in a DID strategy to compare World Bank IBRD/IDA lending before and after the crossover to regions with varying initial levels of overall population-level neonatal measurements, infant, and under-five child mortality. Results are not sizeable and therefore we did not include these as a separate table. The results remain qualitatively similar in that there are no significant interactions between population measures of neonatal, infant, and child mortality with crossover.

## Discussion

This article examines the correlations between IDA transition and World Bank financing to the infant and under-five child needs in a geographically limited context. This research informs an important policy question because the transition away from concessional funding mechanisms will result in difficult tradeoffs allocating limited resources. Recent research finds evidence that donor exits can affect human resources for health, service delivery, medicines, and technologies [7]. The value of this research is especially important in the SDG era in understanding how donor exits could derail progress in health improvement [7]. Our results indicate that there may well be some tradeoffs between graduation from concessional aid and the SDG policy agenda to "leave no one behind" [32]. We find that the alignment of World Bank commitments to regional child needs appears to have diminished after Nigeria crosses the IDA threshold. Conversely, the alignment of World Bank commitments to infant needs seems to have increased. However, these results are statistically insignificant and therefore provide inconclusive evidence.

There are limitations to what can be learned by utilizing a difference in difference approach to compare pre- and post-crossover differences across regions in WB commitments. Most notably, the small sample size could limit the study's statistical power and its ability to detect a positive effect when one is to be detected. To partially deal with this issue, we included in our model-clustered standard errors at the region-year level. Additionally, the DHS data is cross-sectional and causal directions of our model cannot be supported. Therefore, the results produced from this study should be interpreted as descriptive and correlation; causation cannot be inferred. Specifically related to the DHS data and the retrospective nature is the concept of age heaping. In retrospective surveys, heaping at a reported age of deaths is common [33]. It is important to note that age at death is disproportionately likely to be reported at 0, 12, 24, 36, 48, and 60 months due to rounding and recall error. Although interviewers ask about the age at death in months of each child who has died, respondents may round this age to 12-month intervals. For example, some deaths reported at 12 months may have occurred at 10, 11, 13, or 14 months. Bias can be introduced to the extent that age at death results in the net transfer of deaths from one age group to another. There is no way to redistribute individual deaths accurately by changing the age at death. Age heaping is unlikely to be correlated with the timing, but it does introduce measurement error, which could attenuate the coefficients toward zero.

Next, our sample's nature makes it difficult to generalize to other countries transitioning in the next decade. Countries will be unlikely to have the status currently awarded to Nigeria of blended. Finally, despite the advantage of data that geographically references World Bank health commitments, the data does not include information about specific project activities. Therefore it is not possible to attribute the health data to infant or child health-specific interventions. Nonetheless, this research is nationally representative and geographically referenced and hence provided an opportunity to calculate a wide range of regional level infant and child health indicators. This work also has the advantage of longitudinal World Bank IBRD/IDA health aid covering 20 years (1995–2014), which provides an opportunity to measure within-sample change over time.

## Conclusion

Without providing conclusive evidence that crossover results in need-based allocation changes, this research offers an important future research path. We have shown how to craft a flexible approach to utilize existing policy and survey data as a way forward to monitor the aid, post-crossover, in relationship to important SDG indicators. These results are directly relevant to policy debates about what we know and do not know about aid in transition and health [5]. The overall evidence is scarce, but our results are compatible with the existing literature that suggests that donor motives are complicated and driven by factors other than the recipient country [2, 5]. As governments desire to develop their economy and move from a low income to lower-middle-income countries, it is essential to examine how this change is likely to affect these governments receiving aid. Looking ahead, the ideas presented in this work could be pursued further to provide a valuable piece of empirical data necessary to develop clear plans to manage and coordinate transition.
